# Applying probability-weighted incubation period distributions to traditional wind rose methodology to improve public health investigations of Legionnaires' disease outbreaks

**DOI:** 10.1017/S0950268820000230

**Published:** 2020-02-19

**Authors:** D. Bays, E. Bennett, T. Finnie

**Affiliations:** Public Health England, Manor Farm Road, Porton Down, Salisbury, SP4 0JG, UK

**Keywords:** Airborne pathogens, legionella, Legionnaires’ disease, wind rose

## Abstract

In the event of a Legionnaires' disease outbreak, rapid location and control of the source of bacteria are crucial for outbreak management and regulation. In this paper, we describe an enhancement of the traditional wind rose for epidemiological use; shifting the focus of measurement from relative frequency of the winds speeds and directions to the relative volume of air carried, whilst also incorporating probability distributions of disease incubation periods to refine identification of the important wind directions during a cases window of exposure, i.e. from which direction contaminated aerosols most likely originated. The probability-weighted wind rose offers a potential improvement over the traditional wind rose by weighting the importance of wind measurements through incorporation of probability of exposure given an individual's time of symptom onset (obtained through knowledge of the incubation period), and by instead focusing on the volume of carrying air which offers better insight into the most probable direction of the source. This then provides a probabilistic distribution of which direction the wind was blowing around the time of infection. We discuss how the probability-weighted wind rose can be implemented during a Legionnaires' disease outbreak, and how outbreak control teams might use it as supportive evidence to identify the most likely direction of the contaminated source from the presumed site of exposure. In addition, this paper discusses how minor adjustments can be made to the method allowing the probability-weighted wind rose to be applied to other non-communicable airborne diseases, providing the disease's probability distribution for the incubation period distribution is well known.

## Introduction

Legionnaires' disease is a type of pneumonia, caused by the inhalation of Legionella bacteria suspended within water droplets. Legionella bacteria occur naturally in fresh water environments, only becoming a health concern when they grow and multiply in water systems with which humans have interaction, such as air conditioning units, cooling towers, hot tubs or hot water tanks [1]. The number of reported cases of Legionnaires' disease has increased annually in England and Wales since 2013, with the number of confirmed cases of 2018 (January–November) being up to 65% compared to 2013 (January–December) [2–7]. Such an increase has highlighted the need for additional ways of quickly locating potential sources in an outbreak.

The use of meteorological records to aid the source location in a Legionnaires' disease outbreak is well established [8–11]; the predominant method of transmission to humans is via the inhalation of contaminated aerosolised water droplets that have been vented from water systems located in high and/or exposed positions and carried by winds. Wind roses have been used by meteorologists as an analytical tool for centuries, with the earliest recorded wind roses being used by 13th century European sailors to display the principle winds on navigational maps [12]. More recently however, wind roses are used to display the fraction of the total amount of time the wind spent blowing in each direction at specific speeds, over a chosen time range, for a given location. As such, they concisely display a large amount of information in an easily interpreted graphical format. For this reason, they have found their way to being used in the field of epidemiology, where investigators use wind roses in attempts to trace the movements of airborne contaminant particles [13, 14]. Where implemented, these wind roses are used to display trends in prevailing winds over suspected periods of exposure [13–15]. However, epidemiological investigators are less concerned with the frequency of wind speeds, compared to where the contaminated particles most likely came from. For investigative purposes, the traditional wind roses also fail to utilise information provided by the disease itself. The usual method is to draw a wind rose for a specific location (e.g. suspected location of infection), over a defined time range (during which infection is supposed to have occurred). The wind rose is then used to investigate possible trends in the wind's behaviour over the supposed period of infection and suggest a potential direction (from the suspected location of infection) in which the source of the pathogen may be located. In some instances, this may be straightforward (i.e. when there is a dominant wind over the entire period), making the evidence which the wind rose presents immediately obvious. However, in other cases, it may be that the wind has behaved erratically over the time range of interest, at which point interpretation of the wind rose may be more difficult. During outbreaks, we are likely to have additional information on the disease from outbreak control teams, such as an approximate time of symptom onset. This would allow for the categorisation of some wind measurements to be more important than others (e.g. winds blowing just prior to a case's symptom onset are very unlikely to have carried the bacteria which infected the case, when compared to winds a few days earlier).

This paper describes how, by changing the focus from the proportion of time the wind spent blowing to the volume of total air blown and incorporating the probability distribution of the incubation period of Legionnaires' disease, the traditional wind rose can be adapted and subsequently used to provide a more efficient epidemiological tool when trying to locate putative sources of Legionella bacteria. More specifically, given the probable location of infection and date and time of symptom onset for a case of Legionnaires' disease, how disease control teams can create a more effective wind rose to use in their investigations by incorporating the distribution of incubation time for Legionnaires' disease into their meteorological data. This change, though small, has the potential to reduce the time spent by public health teams investigating possible sources during an outbreak, leading to a reduction in further infections; this has the secondary benefit of reducing the costs of the investigation. Additionally, the proposed changes will also remove the need to distinguish between the different wind speeds measured, which helps to simplify the interpretation.

## Methods

### Data

The standard construction of a wind rose requires meteorological data, in the form of wind speed and wind direction, to be recorded at regular intervals. For the construction of a wind rose for epidemiological purposes, the data for the days preceding an infection or an outbreak need to have also been collected near the presumed location where an individual was infected (typically the case's home location). Such data are typically available from national meteorological offices.

### Time increments

Regarding the example explored in the following, we shall be using meteorological data from the UK's Met Office. The measurements used in this paper are therefore taken on an hourly basis, in line with the Met Office recording practices.

### Incubation period

Due to the variation in health among individuals within the general population, as well as variations in received Legionella doses, the incubation period of Legionnaires' disease can also vary. Within this paper, we model incubation time using a two-parameter *γ* probability density function, with shape parameter *a* = 4.96 (95% confidence interval 3.82–6.32) and scale parameter *b* = 1.27 (95% confidence interval 0.99–1.68). These parameters were obtained from [16], where they were derived from the maximisation of log-likelihoods on data from the Melbourne outbreak in April 2000.

**Fig. 1.**
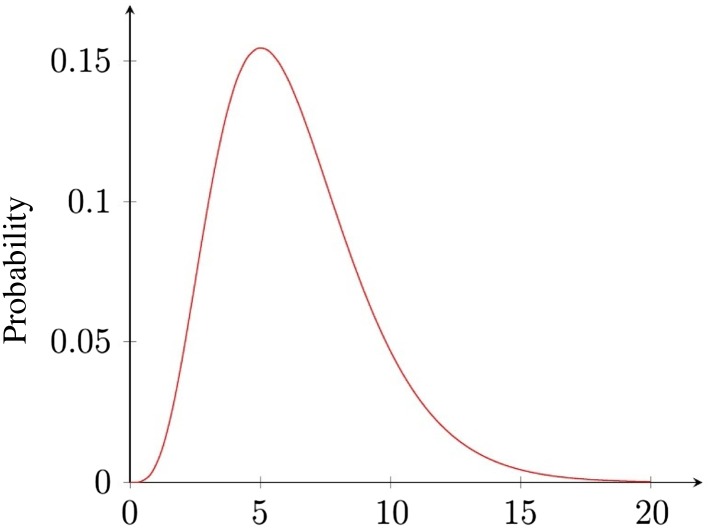
Length of the incubation period (days).

We have also chosen to use 20 days as the upper limit to our incubation period, and so truncate the distribution at this point. The range of 0–20 days, although being much longer than the 0–10 day period quoted by the World Health Organization [17] (WHO), captures 99.95% of the distribution according to the outlined parameterisation. Thus, this range provides what we deem to be a suitably compact time scale for a high percentage of the total distribution. Were we to restrict this to the 10 days suggested by the WHO, this would only capture 89.6% of the distribution, omitting a substantial part of the distribution's tail. Figure 1 depicts the probability distribution function as described here.

### Time of infection

We shall be proceeding under the widely-accepted assumption from the epidemiological back calculation that if we are given a time for symptom onset, we can use the above probability distribution for the incubation period [16] to deduce a probability distribution for the time of infection. Explicitly, if we have an infected case whose symptoms became observable at time *T*, and we have a probability distribution for the incubation period described by the function *f*(*t*), where *t* is the time since infection, then we may assume that the probability distribution for the time of infection takes the graphical form *f*(−*t*), where −*t* represents time prior to symptom onset (i.e. probability of infection occurring at time *T* − *t* is *f*(*t*)). So, for our incubation period described above, we have a probability distribution for the time of infection which takes the exact same form (see Fig. 1), but the *x*-axis would show the range [0, −20] instead, representing days before symptoms became prevalent.

### Wind behaviour

Unless the system we choose to implement is taking meteorological measurements at a near continuous rate, we are required to make some assumptions as to how the wind behaves between consecutive measurements. For the work in this paper, we assume that once a measurement has been recorded, the wind remains constant in that state until the next measurement has been taken (note that Met Office recordings in fact represent hourly averages [18]). Additionally, we shall assume that the wind at the case's location is identical to that recorded at the nearest measuring station.

### Pathogen concentration by varying wind speed

Finally, for further implicity, we shall assume that the concentration of the airborne Legionella bacteria is in a state of stable equilibrium with the immediate surroundings of the source, which is also inexhaustible. Furthermore, we shall assume that in the instance that the Legionella concentration is perturbed from equilibrium, the source of the bacteria has a positively infinite restabilisation rate. This then describes a system where the faster the wind blows around the polluting source, the more the Legionella bacteria are carried by the wind in the direction it is currently blowing. In fact, the relationship between the wind speed and concentration of Legionella bacteria transported downwind is linearly correlated.

Evidence suggests that the distance legionella bacteria can travel as contaminated aerosols (while still surviving long enough to be able to infect a host) is somewhere in the range of 6–12 km [19]. This can therefore provide somewhat of a maximum distance investigative teams might need to travel in search of possible sources; although, this is not a hard limit and so in reality sources may lie outside of this range [19].

## Construction of probability-weighted wind roses for an example case

To demonstrate the construction of a probability-weighted wind rose, we assume a scenario using simulated case data.

### Case outline

Case X reported becoming ill (symptom onset) at 0:00 on 1 June 2018. Case X lives in the postcode area M5 3EX, where they spend most of their time. Case X states that they made no significant travel outside of Manchester during the previous 2 weeks. Case X has been unemployed for some time, and as such, there is no additional workplace to consider as a location of the infection.

The working hypothesis is that the Legionella-contaminated aerosols have been carried by the wind from some nearby source. The source is not inside the case's home, as sampling results are negative, but the source may be relatively close by (a few miles [19]), since there is no history of significant travel outside of Manchester during the preceding 20-day period where we assume exposure occurred.

### Data collection

We take the case's postcode as our focal point, since this is where our case has spent the most time, and therefore most likely to have been exposed. We then identify the nearest recording Met Office station, to give the best description of the weather at the case's postcode. This can be done by using a coordinate system which permits the Euclidean metric to find the recording station whose coordinates minimise the Euclidean distance (in this example we have used British National Grid coordinates due to the flat representation of the UK they provide). In this instance, the nearest Met Office station is located at Rostherne.

To assemble the relevant data, we take the hourly measurements from the weather station (Rostherne) for the day of symptom onset, as well as the preceding 19 days for a total of 480 hourly measurements. For the following calculations, the data are taken to be in the form (most recent reading first):
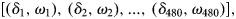
where *δ*_*n*_ and *ω*_*n*_ represent measurements for the wind direction and wind speed *n* hours before symptom onset, respectively.

### Probability coefficients

We then weight the hourly readings following the idea set out in section ‘Time of infection’. Given a function describing the probability distribution for the incubation period, we may describe the probability distribution for the time of infection prior to the time of symptom onset. We shall be using our previously defined *γ* distribution (with shape parameter 4.96, and scale parameter 1.27) to calculate our probability weighting.

Each hourly measurement is weighted by the cumulative probability that our case was infected in the hour preceding our current measurement. This implies that measurements taken at times consistent with more likely incubation periods [20] receive a higher weighting. For instance, a measurement taken an hour before symptom onset would be weighted particularly low, because by our probability distribution for the incubation period, it is highly unlikely the case would show symptoms an hour after exposure. Whereas, measurements taken around 6 days before symptom onset would receive a much greater weighting.

We calculate our probability weighting using the two-parameter *γ* cumulative distribution function, with the shape and scale parameter as defined above. Therefore, if *G*_c_(*t*) is our *γ* cumulative distribution function for time *t*, then we calculate the weight for the *T*th measurement (i.e. the probability of infection occurring in the time range [*T* − 1, *T*]) through the following formula:
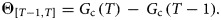


However, it should be noted that the scale and shape parameters as described in [16] parameterise the distribution on a scale of days. As we wish to calculate our weights on an hourly basis, we scale accordingly:
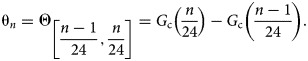


So, *n* will therefore assume integer values in the range [1, 480]. Taking this into account, we then move systematically through the list, weighting our data via the above formula, producing a new list in the form as follows:
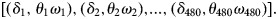


### Drawing the probability-weighted wind rose

Once data are weighted appropriately, they must be reorganised into a form which allows the probability-weighted wind rose to be drawn. To do this, we recall that the *δ*_*n*_ just assumes values describing the wind direction during each measurement. Hence, we could implement an algorithm which would build a list of zeros (one zero for every value *δ*_*n*_ can take), then run through our list of weighted data, and sum the weighted wind speeds which have corresponding values of *δ*. For instance, for our current case, we have taken our data from the Met Office which categorises wind direction through the use of a 16-point compass (i.e. the *δ*'s in our data take values N, NNE, NE,…, NW, NNW). Thus, our algorithm gives us the following form:
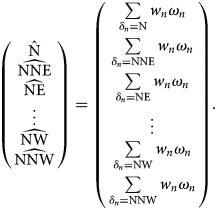


We now produce the probability-weighted wind rose using this method, utilising the Bokeh Python package [21] for the actual visualisation. We have also included the traditional wind rose for the same location and period for comparison.

## Results

**Fig. 2.**
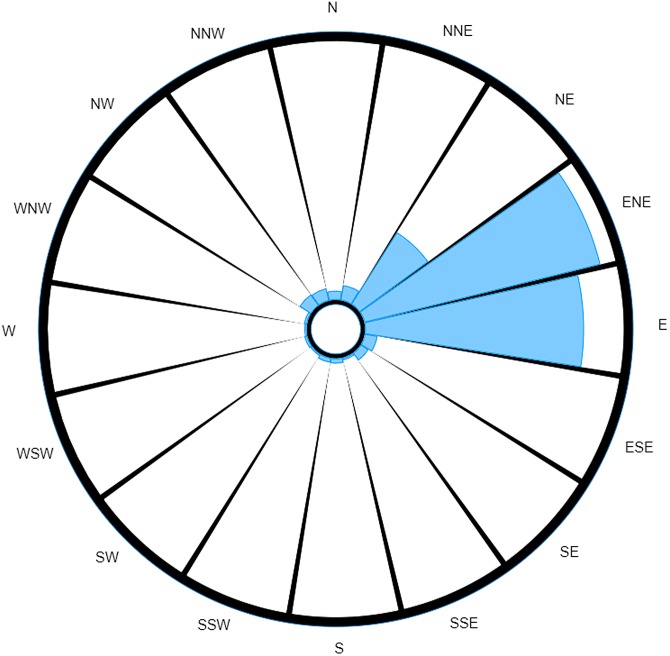
Probability-weighted wind rose for M5 3EX with data taken over a specified time range.

Even though the dominant trend remains, these two wind roses tell two very different stories. The most noticeable difference between the two is how the weighted wind rose (Fig. 2) clearly identifies a narrow range of where the source could be located; being most likely ENE away from the case's home, closely followed by E, and a markedly smaller probability of it originating from NE. The probability for all other directions has been reduced to almost insignificant levels (although, in reality, the lesser readings are unlikely to be of prime interest to investigative teams; hence, their variability will less likely impact the actions of ongoing investigations).

**Fig. 3.**
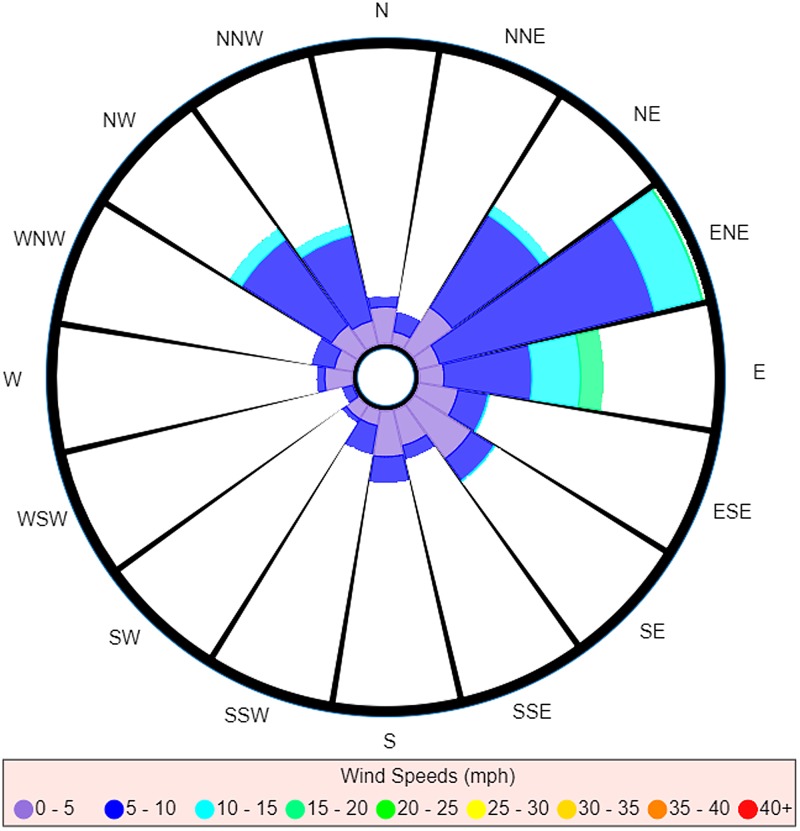
Traditional wind rose for M5 3EX with data taken over a specified time range.

Whereas a strict interpretation of the traditional wind rose (Fig. 3) would still suggest ENE as the most probable direction of the source, we see the reading for E has decreased to such an extent that it now seems to be approximately equal to NE. The readings for NW and NNW are also much more prominent, to the point of being comparable to the readings for NE and E.

This example also highlights another limitation of the traditional wind rose: comparison between similar readings is a more difficult task when dealing with the traditional wind rose. For instance, attempting a comparison between wind directions is not just a matter of comparing total area, for you also have to take account of the different wind speeds that were measured as well as the times these measurements were taken (which is not discernible once the data have been converted to a wind rose). Consider the comparison between NE and E in Figure 3. At a glance, the total areas for both of these directions look roughly equivalent, and so one may be tempted to say it was equally probable to have come from either direction. However, when one takes into account the differences in wind speeds measured for each direction, it is clear that a larger volume of air would have been carried along winds from the east (recall wind's direction reflects direction of origin), and so E would in fact be the most probable direction out of the two (neglecting the discussed effect that timing has on the likelihood of pathogens being carried on winds).

The probability-weighted wind rose removes this source of possible confusion by normalising the recordings (as previously described), such that the magnitude of the displayed measurement is directly proportional to the probability that the causative source is located in each of the directions.

### An extreme example

To further illustrate how the introduction of the probability weighting method improves upon the traditional wind rose, we present an extreme scenario.

**Fig. 4.**
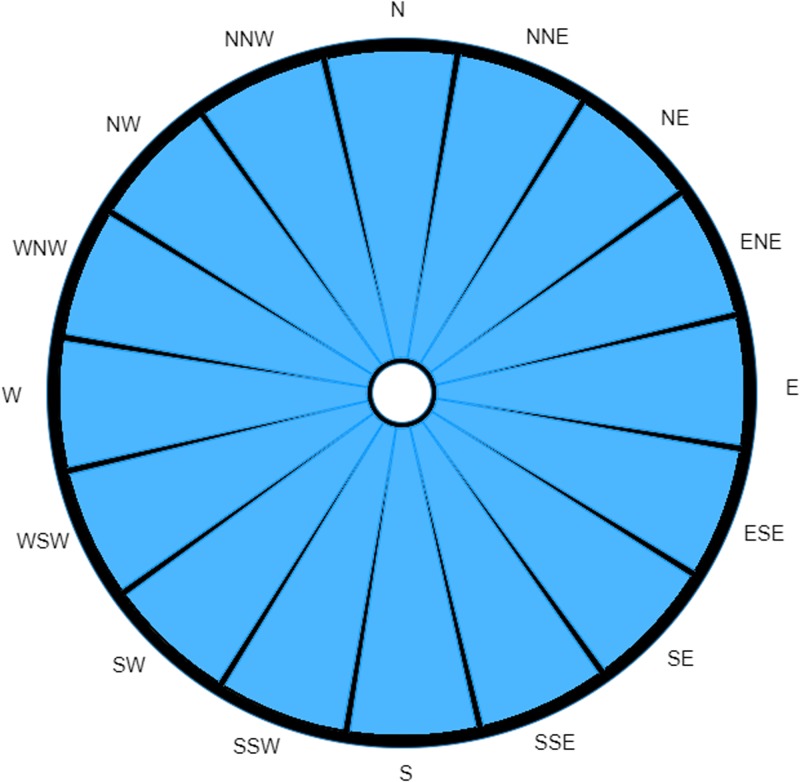
A traditional wind rose for extreme case over a 20-day period.

A case of Legionnaires' disease has been reported without significant travel over the past 20 days. Data from the Met Office station closest to the case's home record the wind over the past 20 days as beginning to blow from the North, maintaining a constant non-zero wind speed and shifting at a constant rate of 18 degrees clockwise per day (completing one full rotation over the 20-day period). As can be seen from Figure 4, the traditional wind rose in this situation is unable to differentiate between any of the directions.

**Fig. 5.**
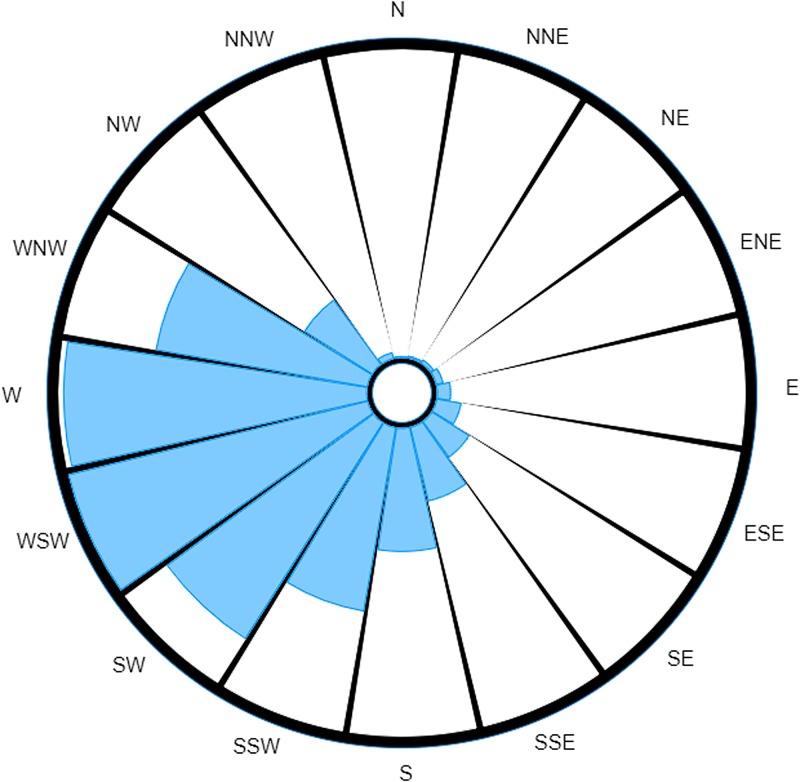
Probability-weighted wind rose for extreme case over a 20-day period.

The traditional wind rose offers investigators no insight into which direction the source would be most likely to be located. However, upon weighting by the incubation period distribution, we produce the probability-weighted wind rose shown in Figure 5.

If we consider the idea of using the readings as a relative guide as to how much time should be spent searching in each direction, this almost instantly disregards any amount of time being spent investigating half of the possible search area for the causative source. It is also immediately obvious that the probability-weighted wind rose in this scenario would provide investigation teams with a most probable direction(s) as to where the source is located.

## Discussion on methodology

### External limiting factors

The main limitations to this method in terms of external influences concern accuracy of measurements, number and placement of measuring stations, and time delay between measurements.

In our example scenario, the data have directional values according to a 16-point compass. However, the method as previously described is flexible such that it can be amended to allow for the use of other data formats.

The input data here are unmodelled, synoptic site data and we have assumed that the behaviour of the wind at a case's location is identical to that at the nearest recording station. This may not however be true given the distance or topography between these two points. A more representative view of the local conditions might be obtained by implementing a modelled dataset, such as the Met Office's Unified Model.

Similarly, for the temporal domain, as with the spatial domain, the wind conditions as reported in the hourly measurements are the average of the wind's behaviour over the preceding hour. As such, outlying events such as lulls and gusts will become obscured. However, as this is a mass-action broad-stroke tool, we believe this to be an acceptable compromise.

### Internal limiting factors

The methodology is also limited due to internal issues which arise from assumptions made about the discrete behaviour of the wind between measurements, assumptions made about the pathogen concentration carried by the wind and a failure to integrate the dynamics of a person's true movements.

We have assumed that after a measurement has been recorded, the wind remains in this state until the next recording is taken. While calculating the wind's exact behaviour between these two points is impossible, methods of interpolation could quite easily be introduced to address some of the limitations caused by such coarse data.

We also assumed that sources of Legionella bacteria exist in a state of stable equilibrium, such that any perturbation results in an instantaneous response returning the system to its state of equilibrium. However, if we try to account for more realistic behaviours, the system becomes more complicated and we are explicitly avoiding resorting to the complexities of a dispersion model in this work. By only considering the wind speed, we are in effect examining the distance a particle could potentially be displaced.

Finally, we do not account for a case's dynamic movements. Use of wind roses becomes limited if the case has undertaken moderate travel over the 20-day period. If travel was made within a confined enough space to discount variation, then this method should still hold. But, as travel distance increases, the probability that the wind conditions the case was exposed to were the same as the conditions recorded near to the case's home address can decrease, and hence reliability can reduce. However, even in instances where significant travel is made, wind roses may still provide some useful information. For instance, drawing a wind rose at each of the main locations visited may still provide investigation teams with some clear information for action.

### Possible further applications

The methodology described here makes no assumptions that limit its use to Legionnaires' disease; so, may be extended to address other airborne contaminants that can be carried by the wind. Although there may be limitations depending on the nature of the organism under study. This method would be best employed for non-communicable diseases with well-known, or well-approximated, incubation periods, such as Q fever, where dust contaminated by infected animals may become aerosolised and carried by the wind, or deliberate release scenarios, with agents such as *Bacillus anthracis*.

## Conclusion

By introducing the weighting of data to traditional wind rose methodology, the focus of public health investigations upon sources of exposure may be refined. This revised method uses the incubation period distribution to reduce the importance of the contribution of those winds less likely to have been carrying the contaminated aerosols that caused the infection. Introducing weighting to the construction of a wind rose is a simple addition requiring minimal additional data, but it has the potential to reduce the time spent searching for the source of infection; with the aim of identifying and treating the source in a timely manner and limiting the number of new cases.
